# Case report: Antibodies to myelin basic protein in a podenco-crossbreed dog with seizures

**DOI:** 10.3389/fvets.2024.1398694

**Published:** 2024-09-30

**Authors:** M. Deutschland, I. Boettcher, M. Höltje, H. Prüss

**Affiliations:** ^1^Neurology Referral Service, Berlin, Germany; ^2^Department of Small Animal Medicine, University Leipzig, Leipzig, Germany; ^3^Institute of Integrative Neuroanatomy, Charité—Universitätsmedizin Berlin, Berlin, Germany; ^4^German Center for Neurodegenerative Diseases (DZNE), Berlin, Germany; ^5^Department of Neurology and Experimental Neurology, Charité —Universitätsmedizin Berlin, Berlin, Germany

**Keywords:** encephalitis, myelin basic protein (MBP), magnetic resonance imaging (MRI), dog, cerebrospinal fluid

## Abstract

A 6-year-old female spayed Podenco-crossbreed dog was presented with an unusual type of focal impaired awareness seizures, including sensory ataxia and postictal rest. Magnetic resonance imaging examination revealed pre- and post-contrast agent T1-weighted bilateral symmetric hyperintensities in the lentiform nuclei and globus pallidus. Repeated cerebrospinal fluid sampling showed lymphocytic pleocytosis. Cerebrospinal fluid immunoglobulin G autoantibodies to myelin basic protein (MBP) were detected by immunofluorescence examination with strong binding to myelinated fiber tracts. The absence of binding to MBP-depleted mouse brains confirmed MBP as an antigenic target. Although the patient had minor seizure episodes every 2 months, and the owners avoided seizure triggers, they refused medical treatment before presenting to the veterinarian. To the best of our knowledge, this is the first description of MBP autoantibody-positive encephalitis in a dog.

## Introduction

In human medicine, the role of autoantibody-mediated encephalitis has profoundly changed the assessment of neurological diseases, and new underlying autoantibodies are continuously being discovered, the most common of which are antibodies against the N-Methyl-D-aspartate (NMDA) receptor and the synaptic protein leucine-rich glioma inactivated-1 (LGI1) (1). These types of autoimmune encephalitis can also occur in veterinary medicine (2). For example, NMDA receptor encephalitis has been identified as a cause of seizures and death in a polar bear (3). Recently, the existence of NMDA receptor antibodies in cerebrospinal fluid (CSF) was described in 3 out of 32 dogs with central neurological disorders (4). Similarly, encephalitis with LGI1 antibodies was confirmed in cats with complex focal seizures and orofacial movements (5). A third type, associated with CSF autoantibodies against the astrocytic glial fibrillary acidic protein (GFAP), was first discovered in dogs with necrotizing meningoencephalitis (6) and later in humans with autoimmune meningoencephalitis and myelitis (7). Recently, an extensive antibody search in CSF and serum demonstrated a neuropil staining pattern on a tissue-based assay compatible with γ-aminobutyric acid-A receptor (GABAAR) antibodies in a young Cavalier King Charles Spaniel dog with refractory seizures (8). Hasegewa et al. (9) found antibodies against the netrin-1 receptor in one cat with suspected hippocampal necrosis in the absence of autoimmunity against LGI1. Importantly, antigenic sequences can show a very high level of homology between several mammalian and human protein targets. To the best of our knowledge, this is the first case report of a dog harboring CSF autoantibodies against myelin basic protein (MBP), thus representing a fifth type of autoantibody-mediated encephalitis encountered in both humans and animals.

## Case description

In May 2017, a privately owned 6-year-old female spayed Podenco-crossbreed dog was presented to the local veterinarian after a week of episodes that included loss of balance for 20 s without loss of consciousness. These events occurred both during daily activity and at rest and were followed by periods of kyphosis, which lasted for minutes. The dog had previously lived in Portugal, but routine blood examinations to detect infectious diseases (*Babesia canis* antibody titer, *Ehrlichia canis* antibody titer, *Leishmania* spp. ELISA, *Hepatozoon canis* PCR, *Filariae* PCR, and *Anaplasma platys* PCR) indicated no exposure. The dog was fed raw meat and a gluten-free diet. The initial suspicion of convulsive syncope was ruled out through electrocardiography (ECG) and cardiac examination, which revealed only minimal mitral valve reflux. Concurrent blood examination revealed minimally elevated serum albumin of 45.3 g/L (reference range: 25–44), increased hemoglobin of 194 g/L (reference range: 150–190), and high-normal blood eosinophilic granulocytes of 0.6 G/L (reference range: 0.04–0.6). Additional low serum thyroxine (T4) level of 0.82 μg/dL (reference range: 1.3–4.5) was corrected with 100 μg levothyroxine–sodium (Forthyron 200 μg, Eurovet Animal Health B.V., the Netherlands) without further examination of serum thyroid-stimulating hormone (TSH). The control T4-serum level was within normal limits. In December 2017, another cluster of seizures similar to those that occurred in May was witnessed by the owner. Repeat blood cell counts revealed no further increase in any blood cell counts, with eosinophils recording lower counts than previously. The non-fasted bile acid serum level was slightly increased (29.6 μmoL/L, reference range: <20).

The dog was referred for further seizure investigation to the Department of Small Animal Medicine, University of Leipzig, Germany. A full examination of the dog including abdominal ultrasound, neurological examination, and serum antibody titers for *Toxoplasma gondii* and *Neospora caninum* revealed no abnormalities. In January 2018, a magnetic resonance imaging (MRI) scan (Ingenia Elition 3.0 T, Philips Healthcare, Best, Netherlands) and CSF examination were performed under general anesthesia.

**Figure 1:**
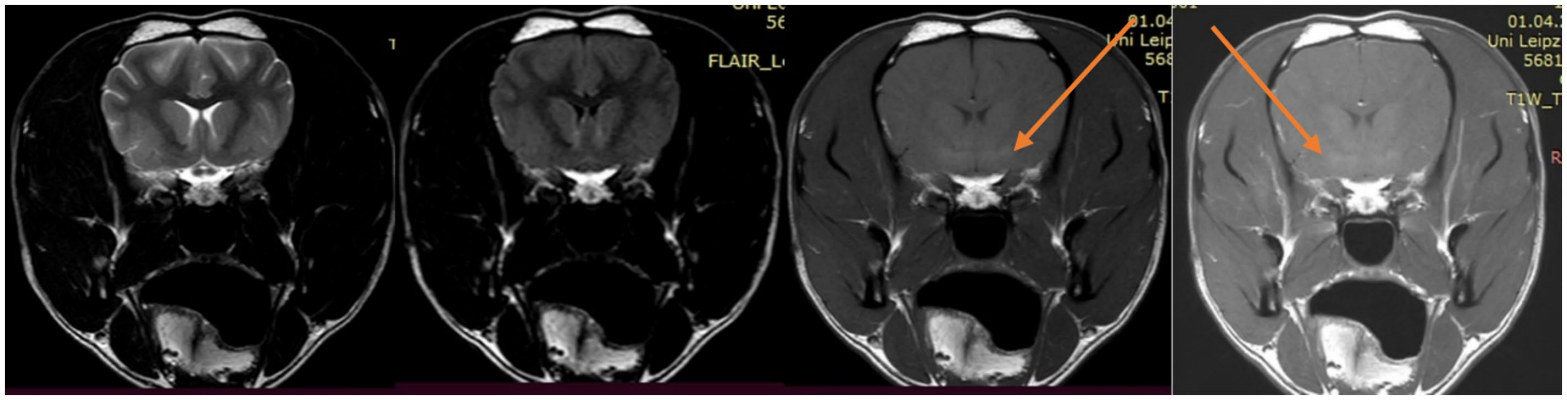
From left to right, transversal planes: T2-weighted (TR 5030 ms, TE 90 ms), FLAIR-weighted (TR 11000 ms, TE 125 ms), T1-weighted (TR 500 ms, TE 20 ms) before and after administration of gadolinium. Note the bilateral hyperintensity in the lentiform nuclei (arrows).

An MRI of the brain revealed bilateral symmetric non-contrast agent-enhancing hyperintensities in the lentiform nuclei and globus pallidus on pre- and post-gadolinium-administered T1-weighted scans, T2-fluid-attenuated inversion recovery (FLAIR) (Figure 1), and diffusion (DWI)-weighted images revealed no abnormal findings.

Differential diagnoses for pre- and post-contrast hyperintense T1- and isointense T2-weighted bilateral lesions of the basal nuclei and thalamus in dogs and humans are portosystemic shunt (due to manganese accumulation and acute hyperammonaemia) (10, 11); acute intoxication with carbon monoxide, methanol, or cyanides caused by impairment of mitochondrial cellular respiratory enzymes (12); hyperglycemia/hypoglycemia, canine copper accumulation (Wilson disease); hypothiaminosis; and autoimmune encephalitis. Tobin et al. (13) showed a high signal only in the caudate head and lentiform nuclei on T2/FLAIR-weighted images, suggesting NMDAR encephalitis. Heine et al. (14) found features of several autoimmune limbic encephalitis corresponding to T2/FLAIR hyperintense signal changes in the medial temporal lobes, including the hippocampus.

The CSF sample examination revealed a medium-graded lymphocytic pleocytosis of 54 cells per μL (reference range: 0–5) with a protein content of 18 mg/dL (reference range: <25). Stained slides were sent to the Justus Liebig University Clinic for Small Animals for further clinicopathological examination and interpretation. The findings included a large number of mainly medium to large lymphocytes with minimally eccentric, round, occasionally notched nuclei. These lymphocytes had a low to medium amount of light to medium basophilic cytoplasm, some of which partly exhibited perinuclear lighting. Single large lymphocytes were suspicious for lymphoblasts.

A few days later, in order to rule out a vascular hepatic disorder, pre- and postprandial serum bile acids were measured, indicating normal hepatic activity.

The initial differential diagnosis was an infection, with suspected causes including viral, protozoal, bacterial meningomyeloencephalitis, meningomyeloencephalitis of unknown origin (MUO), or multicentric central nervous system (CNS) lymphoma. CSF sampling was repeated under sedation after 7 weeks. No treatment was given in between. CSF pleocytosis rose to 125 cells per μL, with a similar distribution of cells: 79% were activated lymphocytes, of which some were in mitosis with heterogeneous appearance (nucleus, nucleus–cytoplasm relation, and size), some cells showed scattered chromatin, and some had basophilic cell plasma. Due to the high suspicion of lymphoma, therapy with prednisolone and lomustine was recommended.

Prior to the initiation of chemotherapy, the owners sought local support for their dog and a second opinion from the Neurology Referral Service in Berlin. An initial examination revealed no neurological abnormalities, with the patient being bright, alert, and responsive. The dog had not displayed any further seizure activity since the last cluster and had not received any medication.

**Figure 2:**
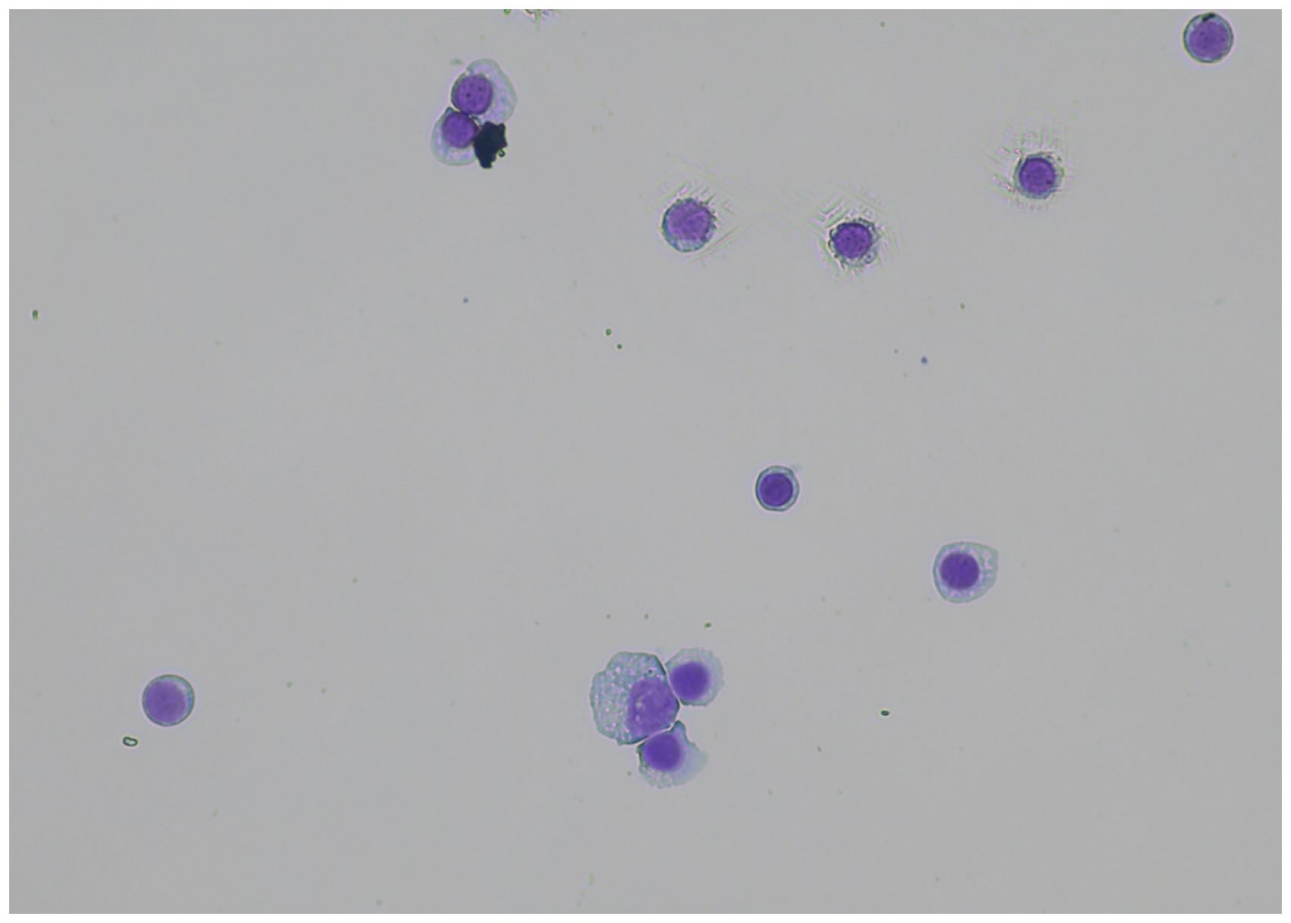
CSF of the patient at the time of the second tap. Note the heterogeneous appearance of the lymphocytes concerning cell size, nucleus–cytoplasm ratio, and appearance of the cytoplasm.

In March 2018, CSF sampling was repeated and confirmed the previous cellular findings (Figure 2). CSF inflammatory parameters such as immunoglobulin A (IgA) 0.5 mg/dL (reference range: <0.1) and C-reactive protein (CrP) 5.0 mg/L (reference range: <1.0) were increased. Additional examination of antibodies in CSF against tick-borne encephalitis virus showed a concentration of 4.3 units/mL (reference range: <63), and the detection of DNA of distemper virus, *Toxoplasma gondii*, *Neospora caninum*, and *Anaplasma phagocytophilum* using the PCR method was negative (Laboklin GmbH & Co.KG, Laboratory for Clinical Diagnostics, Bad Kissingen, Germany).

As the majority of differential diagnoses were excluded by the blood examinations (e.g., portosystemic shunt) or seemed unlikely (e.g., repeated intoxications over a period of 3 months), an unusual case of CNS lymphoma remained possible. On the other hand, the patient showed very little progression since the first MRI and CSF findings, and the lymphatic cell population seemed rather heterogeneous, which made neoplasia less likely. Therefore, 50 μL of CSF was sent for further examination in tissue-based assays to rule out an unusual chronic form of encephalitis, such as the NMDA receptor encephalitis previously described in a polar bear (3).

**Figure 3:**
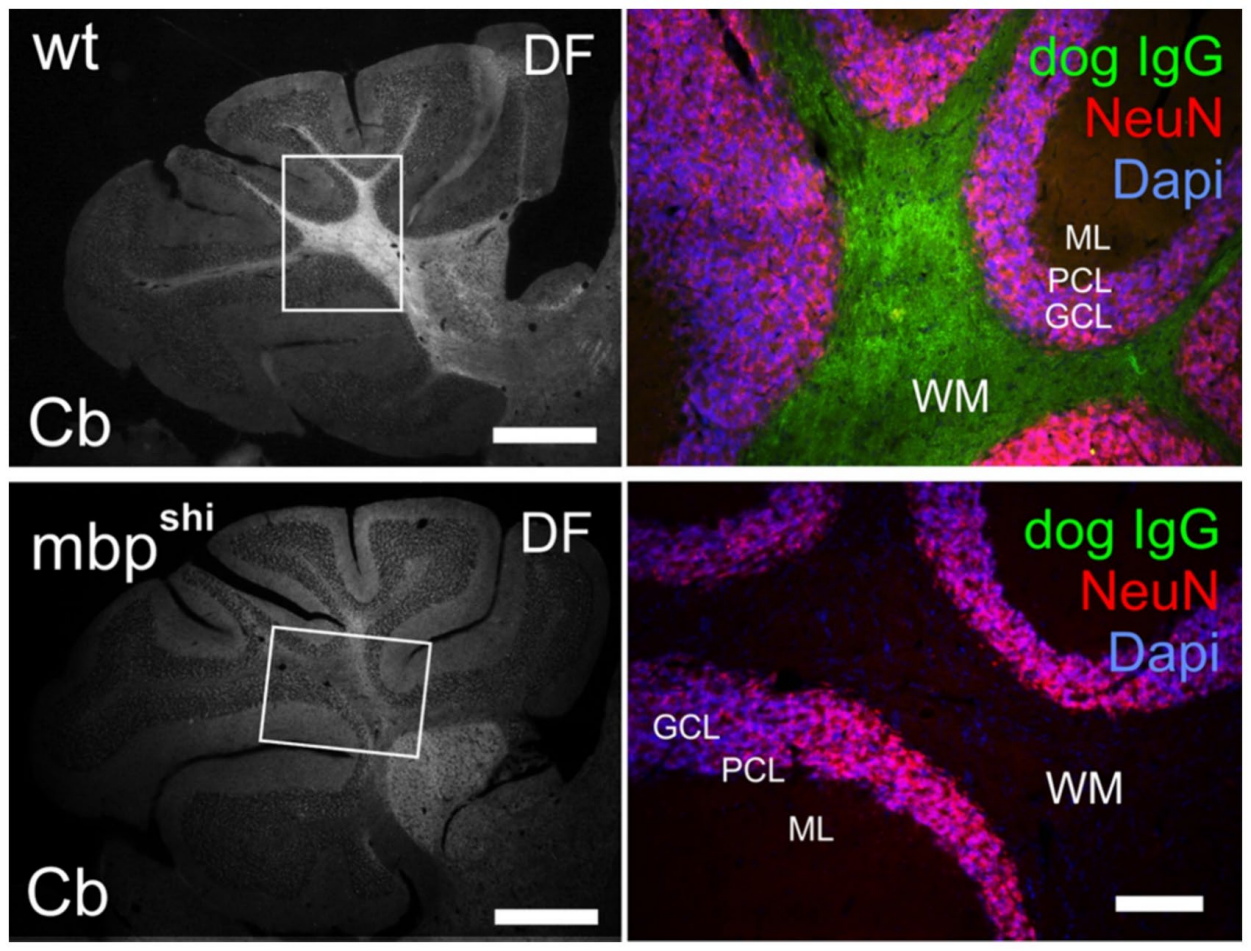
(Top) Immunofluorescence of dog IgG antibodies (green) on a 20-μm paraformaldehyde-fixed mouse cerebellum section showed strong binding to myelinated tracts in the white matter (double-labelling with DAPI for cell nuclei in blue and with the neuronal marker NeuN in red) (Bottom). The strong binding to axonal fiber tracts was completely absent in shiverer MBP null mutation (mbpshi) littermate mice. Bars represent 500 μm (left) and 100 μm (right). Cb, cerebellum; DF, dark-field illumination; GCL, granule cell layer; ML, molecular layer; PCL, Purkinje cell layer; WM, white matter; wt, wild type.

Using a well-established methodology of indirect immunofluorescence on murine brain sections (1, 15, 16), we incubated 20-μm cerebellum sections with undiluted CSF overnight at 4°C, washed with phosphate-buffered saline (PBS), and visualized after 2-h incubation at room temperature using fluorescein isothiocyanate (FITC)-labeled affinity-purified rabbit anti-dog IgG (1,400; Dianova, #304-095-003). The dog’s CSF IgG showed strong binding to myelinated fiber tracts (Figure 3, top), indicating MBP as a likely antigenic target. Indeed, MBP specificity was confirmed using MBP null-mutation shiverer mice, a mouse strain harboring a naturally occurring autosomal recessive loss of function mutation of the MBP gene (17), in which the antibody binding was completely lost (Figure 3, bottom).

## Outcome

The owners declined chemotherapy or corticosteroids for the patient. The dog was examined regularly by the local veterinary surgeon, and 84 months after the first episode, only minor seizures of a few seconds occurred approximately every 2 months, frequently in stressful situations (e.g., physiotherapy and Christmas Eve), and with recognizable phases including an aura and postictal relaxation, without loss of consciousness.

## Discussion

MBP autoantibody-associated encephalitis has recently been described in a human patient with suspected multiple sclerosis (MS), CSF pleocytosis, and high levels of MBP antibodies (15). Clinical improvement with immunotherapy in this patient paralleled the disappearance of antibody titers, suggesting that the antibodies may have contributed to the disease.

MBP autoantibody-associated encephalitis could be an unrecognized form of canine autoimmune encephalopathy and could enable a distinction between lymphatic CNS neoplasia and lymphocytic pleocytosis due to inflammatory disease. In the described patient, neoplasia is very unlikely because the first symptoms appeared almost 6 years ago and the dog survived without any treatment. The median survival time of human patients under treatment for primary CNS lymphoma was only 42 months (18). In addition, a meta-analysis from the United States proved that the 5-year survival rate remains low at 33% (19). In 18 dogs, the median survival time was 171 days (range: 1 to 1942 days), with 15 dogs receiving various treatments, e.g., surgical decompression, chemotherapy, and radiotherapy (20).

Receptor autoantibody encephalitis is well-recognized in humans (1). Autoimmune diseases of the brain found in humans often have equivalents in veterinary medicine. For instance, necrotizing meningoencephalitis (NME) in pugs is associated with dog leucocyte antigen (DLA) class II, similar to how MS in humans is associated with specific human leucocyte antigen (HLA) class II variants (21). Furthermore, hippocampal sclerosis in humans and cats shares similar features (22). Stafford et al. (4) proved several targets of their study, such as the effectiveness of immunofluorescence assays designed for human use in the detection of NMDAR antibodies in canine CSF. Additionally, they could prove that sequence homologies between dog and human protein targets of each antibody are between 94 and 99%. Unfortunately, only two of the three dogs with NMDAR antibodies met the inclusion criteria for autoimmune antibody encephalitis based on MRI changes and altered CSF results. The two qualifying dogs were poodles with signs of MUO. The third dog, a French Bulldog, did not show any abnormalities on brain MRI and CSF results, despite clinical forebrain and brainstem signs. Previous steroid treatment may have obscured the findings at the time of the investigation. Hemmeter et al. (23) could not find specific neural antibodies using mouse and human target antigens in 58 dogs with epilepsy of unknown cause and dyskinesia of unknown origin and 57 control dogs. The patient described here may present with a special form of encephalitis with paroxysmal forebrain signs of focal impaired awareness (minimal absence) seizures, bilateral MRI findings, and a continuously elevated CSF lymphocyte count. Due to the normal results of the previous examination and the inconclusive findings of the CSF pleocytosis, autoimmune antibody encephalitis was the most likely diagnosis, although it had never been proven before in canines. The specificity of indirect immunofluorescence was determined through random testing of canine and vulpes CSF.

Immunosuppressive treatment in the case of autoantibody encephalitis has been considered the “gold standard” in humans, often using corticosteroids alone or in combination with immunoglobulin therapy or plasmapheresis (24). In dogs with the diagnosis of meningoencephalitis, steroids such as prednisolone at 2 mg/kg body weight once daily have proven effective. Other immunosuppressive agents such as lomustine, cyclosporine, cytarabine, or leflunomide may be effective (25–28). In the patient described here, medical treatment was declined after intense discussion with the owners, although the seizure activity has never been completely resolved. Stress avoidance, careful use of anti-ectoparasites, and emergency seizure treatment such as diazepam were discussed. It is still debated why the disease never progressed to an increased number of cluster seizures or altered mental status. Ongoing close contact with the owner may provide insight, and a postmortem histological examination may offer further information in the future.

## Limitations

Due to the patient’s relatively good health and low seizure frequency, it is possible that the dog will live for several more years. Consequently, further histopathological examination of the brain may not be available in the near future. Similarly, we lack further CSF and imaging follow-up, as the owners do not want close monitoring of the CSF or repeated MRI examinations due to the risks tied to anesthesia. The relevance of the lesions found on MRI examination concerning the development of the seizures remains speculative without EEG confirmation. In principle, the localizations addressed may show symmetrical hyperintensities on T1- and T2-weighted MRI sequences in the context of different forms of epileptic seizures. These are often associated with genetic-metabolic diseases (29, 30). It is also unclear why the MRI examination revealed pathological signal increases mainly in the gray matter, while there was a concurrent elevation in antibodies directed against MBP. A possible explanation could be the uneven distribution of MBP within the brain, although one-tenth of the MBP present in the white matter is distributed within the cerebral cortex (31).

## Data Availability

The raw data supporting the conclusions of this article will be made available by the authors, without undue reservation.

## Glossary

MBP: Myelin basic protein
MRI: Magnetic resonance imaging
CSF: Cerebrospinal fluid
NMDA: N-methyl-D-aspartate
NMDAR: NMDA receptor
GABAAR: γ-aminobutyric acid-A receptor
LGI1: Leucine-rich glioma inactivated-1
GFAD: Glial fibrillary acidic protein
DCC: Deleted in colorectal carcinoma
ECG: Electrocardiography
T4: Tetraiodothyronine, thyroxine
TSH: Thyroid-stimulating hormone
FLAIR: Fluid-attenuated inversion recovery-MRI-sequence
DWI: Diffusion-weighted magnetic resonance imaging
MUO: Meningomyeloencephalitis unknown origin
CNS: Central nervous system
IgA: Immunoglobulin A
CrP: C-reactive protein
DNA: Deoxyribonucleic acid
PCR: Polymerase chain reaction
PBS: Phosphate-buffered saline
IgG: Immunoglobulin G
FITC: Fluorescein isothiocyanate
CNS: Central nervous system
MUO: Meningoencephalitis of unknown origin
MS: Multiple sclerosis
NME: Necrotizing meningoencephalitis
DLA: Dog leucocyte antigen
HLA: Human leucocyte antigen
EEG: Electroencephalography
